# The "Christian World" and Consumption Sanatoriums

**Published:** 1910-08-13

**Authors:** E. W. Alabone

**Affiliations:** Lynton House, 12 Highbury Quadrant, N.


### THE "CHRISTIAN WORLD" AND CONSUMPTION SANATORIUMS.

*To the Editor of* The Hospital.

Sir,—In your issue of June 11 you openly accused me of the very serious offence of making "gross misstatements" in the *Christian World* in regard to the open-air treatment of consumption, suggesting "ignorant and malicious prejudice." You also referred me for my instruction to Messrs. Latham and Garland's book "The Conquest of Consumption," which I had already perused and annotated. My annotations exactly coincide with the opinion of Mr. Karl Pearson, expressed in a letter published in the *British Medical Journal* of June 18, in which he proves Messrs. Latham and Garland to have made "gross misstatements," and recommends the immediate withdrawal of "The Conquest of Consumption" from circulation. Beyond this, Messrs. Latham and Garland's inaccuracies are of no interest to me. I fully defended my position in a letter published in the *Christian World*, June 23; since that date letters from medical men and others have appeared, entirely corroborating my statements and upholding my views. As you have not already done so, I must ask you kindly to contradict the assertion you have made against me, which was utterly unfounded, and calculated to do me serious injury.—Yours truly,

Lynton House, E. W. Alabone.

12 Highbury Quadrant, N.,

August 7, 1910.

[Our opinion on the question of sanatorium treatment has been fully expressed in the articles which have appeared in The Hospital, and we see no reason to contradict any of our assertions.—Ed. The Hospital.]

